# Seasonal Changes in Trace-Element Content in the Coat of Hucul Horses

**DOI:** 10.3390/ani12202770

**Published:** 2022-10-14

**Authors:** Karolina Jachimowicz-Rogowska, Jadwiga Topczewska, Wanda Krupa, Marcin Bajcar, Małgorzata Kwiecień, Anna Winiarska-Mieczan

**Affiliations:** 1 Department of Bromatology and Food Physiology, Institute of Animal Nutrition and Bromatology, University of Life Sciences in Lublin, Akademicka Str. 13, 20-950 Lublin, Poland; 2 Department of Animal Production and Poultry Products Evaluation, College of Natural Sciences, University of Rzeszów, Zelwerowicza Str. 4, 35-601 Rzeszow, Poland; 3 Department of Animal Ethology and Wildlife Management, University of Life Sciences in Lublin, Akademicka Str. 13, 20-950 Lublin, Poland; 4 Department of Bioenergetics, Food Analysis and Microbiology, College of Natural Sciences, University of Rzeszów, Ćwiklińskiej St. 2D, 35-601 Rzeszow, Poland; 5 Department of Animal Nutrition, Institute of Animal Nutrition and Bromatology, University of Life Sciences in Lublin, Akademicka Str. 13, 20-950 Lublin, Poland

**Keywords:** trace element, horses, season, nutrition

## Abstract

**Simple Summary:**

The proper development and health of the body is determined by the correct concentration and proportion of minerals in tissues, body fluids, and also in the coat. Due to its structure, the coat is an excellent biological material for assessing the accumulation of metals in the animal’s body over a long period of time. Changes in the mineral profile can occur under the influence of various factors, such as age, breed, the quality of feed consumed, and the season in which the sample was obtained. The purpose of this study was to evaluate the effect of seasons on the content of selected trace elements in the hair of Hucul horses kept in semi-natural living conditions. It was found that there were differences in the levels of the trace elements studied according to the season. In addition, positive correlations was shown between the content of iron and manganese as well as iron and aluminium. The occurrence of variability not only within seasons but also between the studied populations indicates the need for further research.

**Abstract:**

The purpose of the study was to evaluate seasonal changes in selected trace elements such as Fe, Cu, Mn, Zn, and Al in the coat of healthy Hucul horses kept in south-eastern Poland in two different facilities and fed with locally sourced feed. The coat for the study was collected from 24 individuals in autumn, winter, and spring. The concentration of elements in the feed was also determined. The date of collection had a significant effect on the concentration of the micronutrients analysed in the coat of Hucul horses. The highest concentration of Zn was found in the coat taken in summer. The coat taken in autumn had the highest concentrations of Fe, Cu, Mn, and Al compared with the other seasons. The highest concentrations of Fe, Mn, and Al were found in fur taken in winter, with the lowest levels of Zn. Positive correlations were found between the content of iron and manganese, iron and aluminium, and manganese and aluminium in the coat of Hucul horses. A clear inter-individual and inter-stable variability was found, which may indicate the need for further research that also takes into account other factors.

## 1. Introduction

Hair is a characteristic mammalian product of the epidermis and, depending on its form and location, can perform many different functions [1,2]. Horse coat is primarily responsible for protecting the skin from various external environmental factors. Due to its thermo-regulatory function, the quality of the coat changes in the annual profile with the seasons. Therefore, the highest growth rate of coat-forming hairs occurs in autumn, which has not been confirmed for mane and tail hair [2]. The quality of hair and coat is believed to be determined, in addition to species affiliation [1], by proper nutrition [3]. Attention is mainly paid to the availability of protein, minerals, and vitamins. However, the nutritional factors affecting growth and the anatomical structure and mineral composition of hair are very complex and can be inter-related. Possible nutritional imbalances that determine normal hair and coat growth include, for example, deficiencies or the availability of certain trace elements or vitamin B and vitamin C [2,4,5,6]. The use of hair in a non-invasive manner to assess the mineral status of animals has received considerable attention in recent years. The key argument is the ease of collection of the material and its storage and the stability of composition [7]. At the same time, results in terms of elemental levels in the blood may be subject to error, since active homeostatic mechanisms compensate for levels in the blood at the expense of reserves in the body [8]. Many authors point out that the advantage of the hair material is also due to the fact that its mineral composition documents the supply of various elements over a longer time profile than is the case, for example, with blood, where changes can occur in a daily profile [9,10]. Hair is a good matrix for the initial assessment of elemental levels [11,12,13]. Furthermore, hair can be collected from the animal non-invasively in vivo, and the collected samples do not require refrigerated storage, which is important when sampling in the field [6,14,15].

It seems that the possibility of conducting a precise and non-invasive evaluation of the mineral status of animals is a particularly interesting alternative to traditional tests for animals living in natural or semi-natural conditions [6,13,16]. Deficiencies of certain trace elements in these animals can be the result of limited availability in the environment and, as a result, negatively affect development, health, or reproductive rates. Regular assessment of the content of important trace elements would allow for quick decisions to implement supplementation and minimise negative effects in animals. On the other hand, the heavy metal content in the hair of wild-type animals is often considered an indicator of environmental quality [13,16,17].

Hucul horses are a primitive breed, kept quite often under natural conditions in their typical environment and fed with roughage. It is distinguished by a better use of less valuable feeds than other breeds, which is important for proper body supply. Additionally, these horses are characterised by low choice and readily eat herbaceous plants avoided by other species. At the same time, as typical herbivores, they obtain trace elements from plants, the mineral composition of which depends on the natural abundance of the soils.

The scientific literature lacks detailed data on the effect of season on the content of elements analysed in the hair of horses kept under semi-natural conditions. The purpose of this study was to evaluate the effect of season on the content of selected trace elements, such as Fe, Cu, Mn, Zn, and Al, in the hair coat of Hucul horses maintained under semi-natural conditions.

## 2. Materials and Methods

### 2.1. Animals

Two stables kept by Hucul horses, located in south-eastern Poland, were selected for the study. The horses were reared from birth in the facilities selected for the study. Twelve mares each were selected for the study. At stud A, the average age was 12 years (range 3 to 20 years), whereas at stable B, it was 19 years (range 11 to 24 years). All mares were fed with roughage. During the growing season, the horses were on pasture, taking forage ad libitum and had constant access to water and salt in the form of a lick. In the off-season, they received hay and haylage and also had constant access to salt in the form of a horse lick and water. These were blocks (Lisal basic licks) with a minimum NaCl content of 95%. Condition assessment was always performed during coat sampling, according to the method proposed by Carroll and Huntington [18]. The horses were in very good condition, rated 3.0–3.5 points on the 5-point BCS scale, indicating optimal condition [19,20]. The mares were protected under the Genetic Resources Conservation Program. This resulted, among other things, in a breeding evaluation by the committee conducted each year and regular foaling. All horses selected for the study were clinically healthy, the herds were under constant care of a veterinarian, and no signs of disease were reported during the study period.

All examinations performed in this study are non-invasive and are routinely performed as part of everyday medical and management procedures. Following the existing law applicable in Poland, based on the Animal Experiments Act of 15 January 2015 (Journal of Laws of the Republic of Poland, 2015, item. 266), non-invasive clinical studies do not require ethical approval.

### 2.2. Fodder and Coat Analysis

Fodder used in horse nutrition was sampled in each season. During the summer season, green fodder was taken at the horses’ foraging sites six times at two-week intervals. Fe, Cu, Mn, Zn, and Al levels were determined in the fodder, and analyses were performed in three independent replications for each sample. The coat samples were taken at the end of each season (summer, autumn, and winter) from the same individuals. Samples were taken around the nape of the neck from under the mane in an amount of approximately 500 mg from each horse. The collected coat was cleaned and prepared for mineralisation. The samples were rinsed using an optimised rinsing process to eliminate contamination before digestion, according to the methodology proposed by Návesník et al. [21]. The levels of selected minerals such as Fe, Cu, Mn, Zn, and Al were determined by inductively coupled plasma optical emission spectrometry. All analyses were performed in three independent replications for each sample. In each season, 24 horses were sampled.

The samples were subjected to high-pressure mineralisation in super pure 65% HNO_3_. Samples (5 g) were weighed and placed in Teflon vessels, which were then filled with 8 mL of nitric acid and tightly sealed. For each group of nine samples, the digestion system rotor was also filled with a blank sample. The samples were digested with the algorithm of increasing temperature applied as specified for biological samples, without exceeding 200 °C; this was carried out using an Ethos One microwave digestion system from Milestone (Sorisole (BG), Lombardy, Italy). The vessels were opened after the mineralisation process had been completed, and the samples with acid had been brought to room temperature. The samples were cooled to room temperature and supplemented with water to a volume of 50 mL. The detection threshold obtained for each element was not lower than 0.01 mg kg^−1^ (with an assumed detection capacity of the measuring apparatus at a level exceeding 1 ppb). Measurements were performed with an ICP-OES spectrometer, Thermo iCAP Dual 6500 (Thermo Fisher Scientific, Schaumburg, IL, USA) with horizontal plasma, and the detection capacity was determined both along and across the plasma flame.

### 2.3. Statistical Analyses

The normality of the distribution of the data in each group was verified using the Shapiro–Wilk test. The results obtained did not have a normal distribution, so calculations were made using nonparametric tests. Mean values and standard deviation (SD) were calculated, and the significance of differences was calculated using the Kruskal–Wallis ANOVA test. Spearman’s R correlations were calculated between the mineral levels in feed and coat and between trace elements for each stable and by season. Comparisons were also made between mineral levels in the coat between individuals according to age. Statistical analyses were performed using Statistica, version 13.3 (Statistica Software, StatSoft, Inc., Kraków, Poland, 2020).

## 3. Results

The average content of trace elements in the feed used in the feeding of Hucul horses is summarised in Table 1.

The results of the analysis of selected micronutrients showed a significantly higher content in the feeds analysed taken up by horses in stable B of Cu, Mn, and Zn in green fodder (*p* < 0.05). In hay, a higher content of Fe and Zn was found in stable A, whereas Mn was found in stable B (*p* < 0.05). However, in haylage, a higher Al content was found in stable B (*p* < 0.05) (Table 1).

Table 2 summarises the levels of trace elements in the intake coat of all Hucul horses, regardless of the intake season. Statistically significant differences were shown between individuals from stables A and B only for Fe (*p* < 0.001) and Mn (*p* = 0.0164).

The results of the analysis of trace-element levels in the coats of Hucul horses according to the season of intake showed statistically significant differences in stables A and B for all elements analysed (Table 3). In stable A, the highest concentrations of Fe were found in the coats of the horses studied in winter, and in stable B in autumn. The lowest concentrations of Fe, Mn, and Al for both stables were observed in coats taken in summer.

Significant differences in Cu concentrations in the coats of Hucul horses were shown between all seasons in horses from stable A, the summer and winter seasons, and the autumn and winter seasons in stable B. The highest concentration of Zn in both the coats of stables A and B horses was determined in samples taken in summer. In the case of Al, the highest concentration in the coats of stable A horses was in samples taken in winter, whereas the highest concentration in the coats of stable B horses was in samples taken in autumn (Table 3).

**Figure 1:**
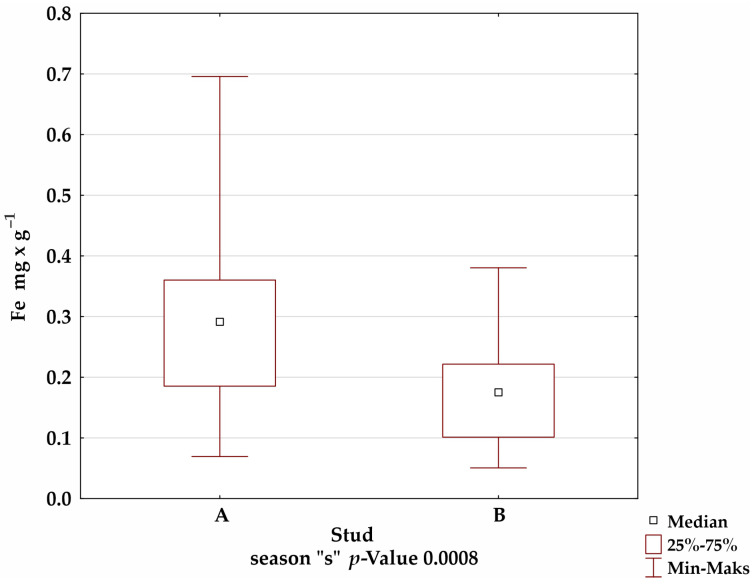

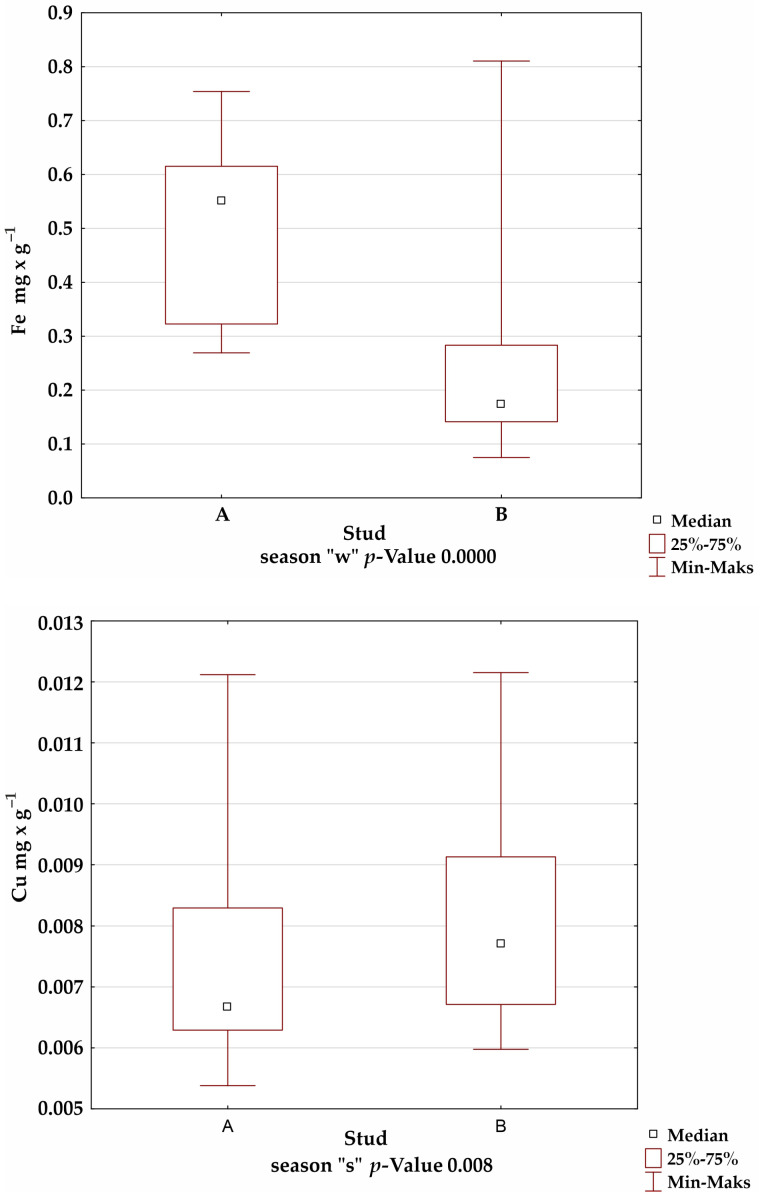

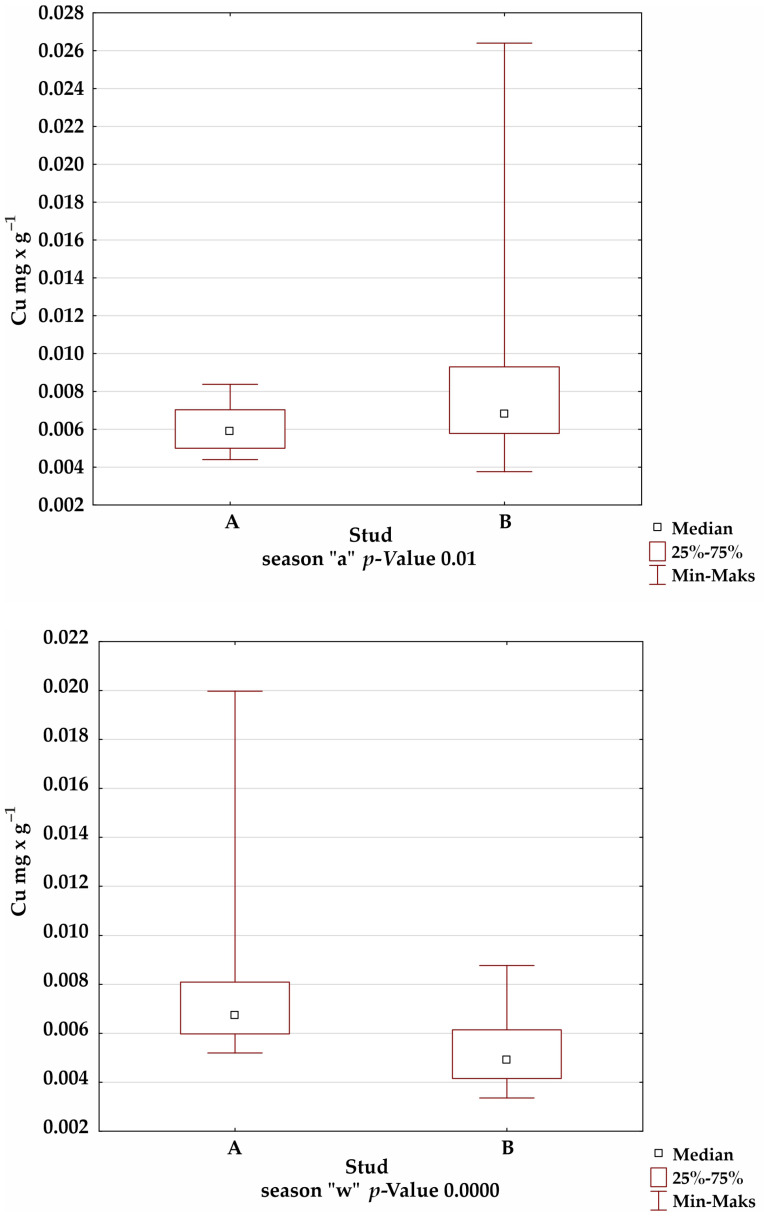

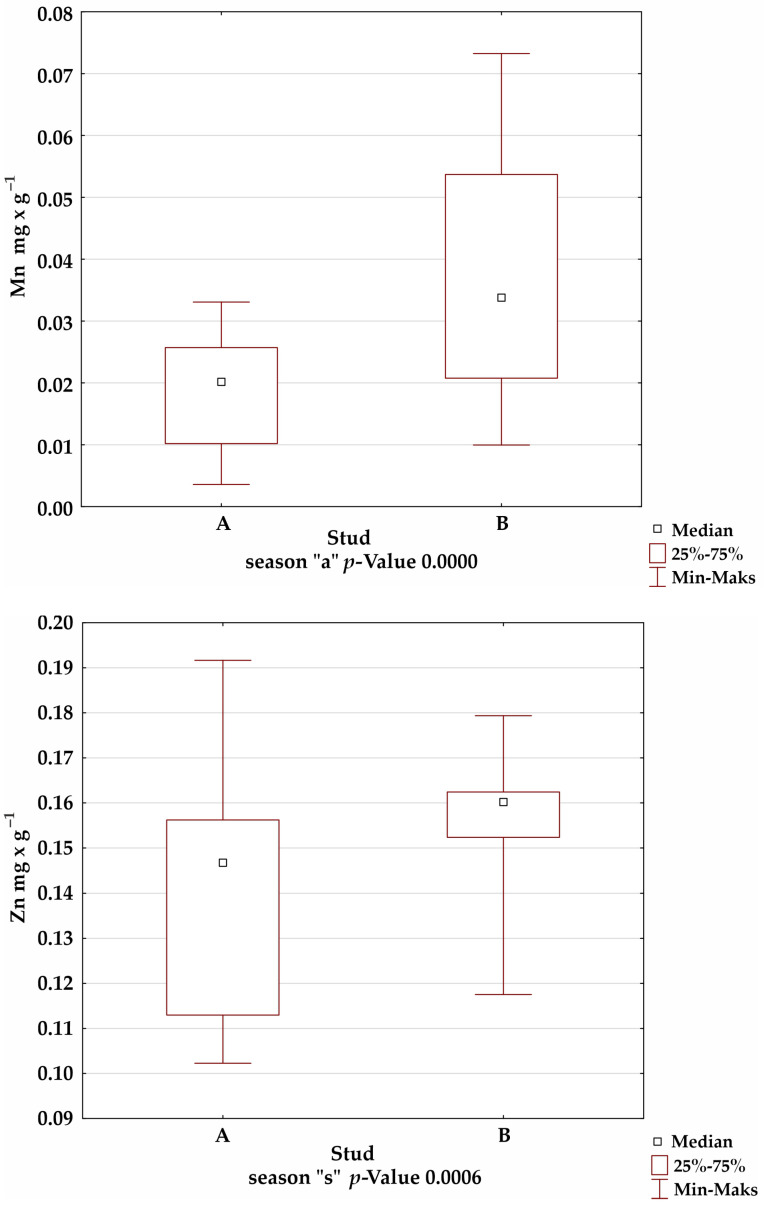

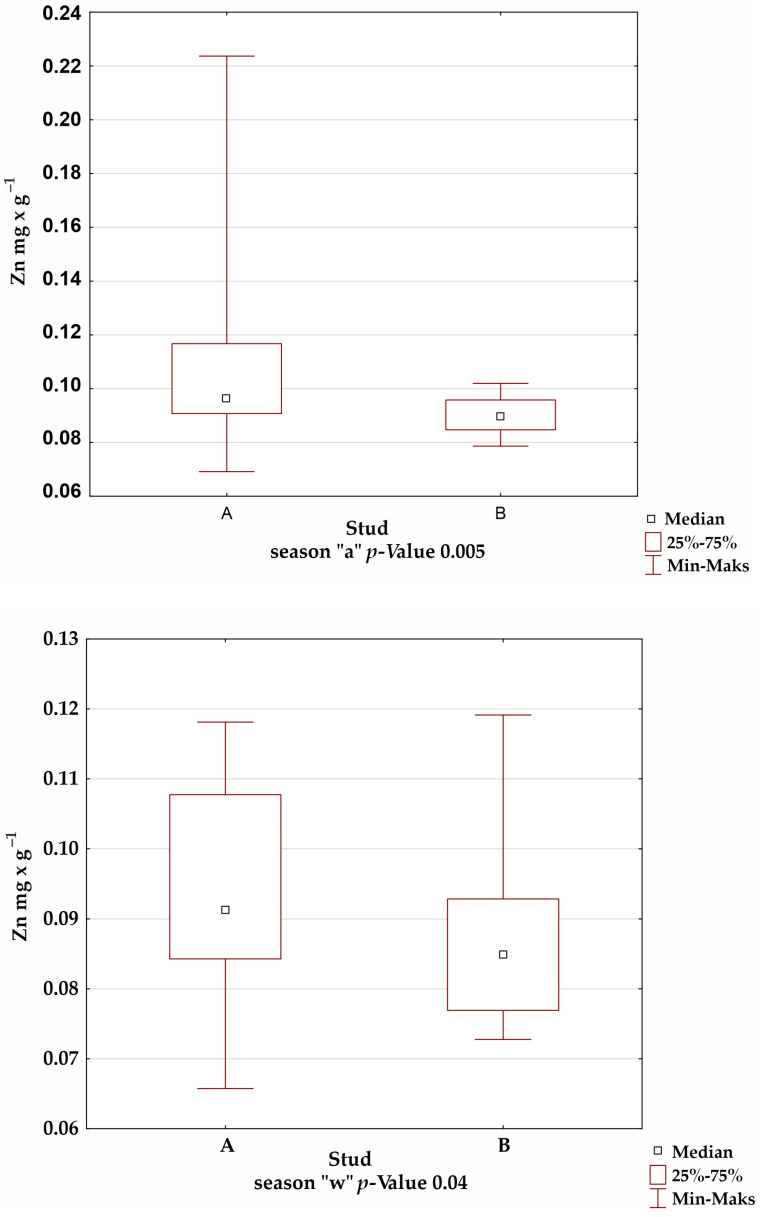

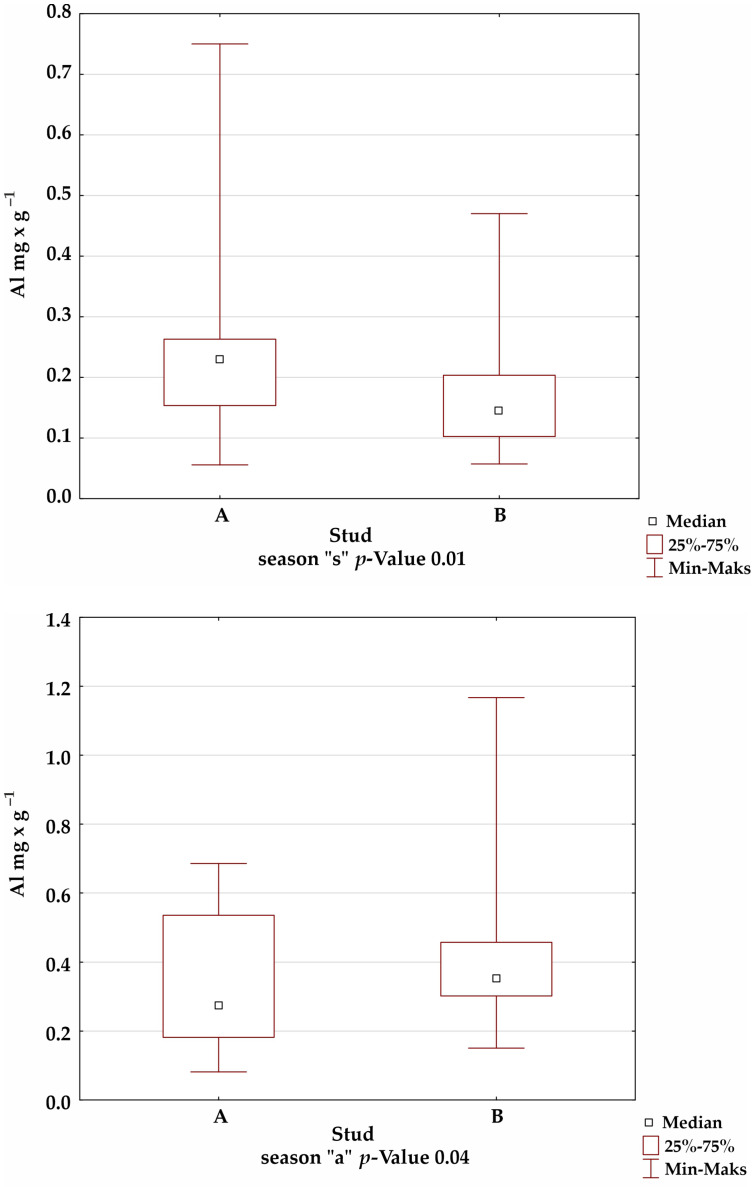

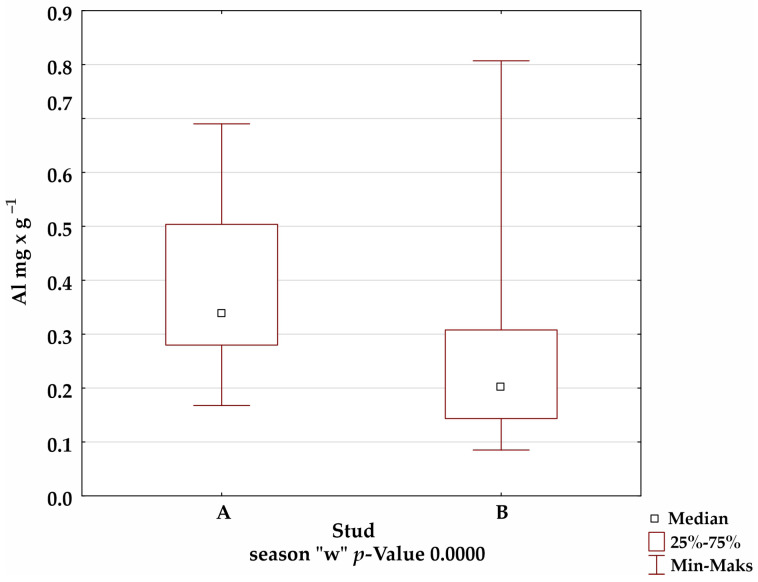
Comparison of trace-element levels between stables in each season, for which the significance of differences was confirmed by Kruskal–Wallis test.

A comparison of the mineral levels between stables A and B confirmed the presence of statistically significant differences for Fe in the summer and winter seasons; Mn in the autumn season; and Cu, Zn, and Al in all seasons (Figure 1).

The correlation between the content of the trace elements analysed in the feed and the coat of the horses is summarised in Table 4. It was found that the correlations were low or medium; in stable A, it was negative for Mn (−0.2693) and positive for Zn (0.2233).

In contrast, correlations were found for all trace elements tested in stable B. The highest negative one (R_s_ = −0.5655, *p*-value 0.0000) was shown for Mn, whereas a positive one (R_s_ = 0.4818, *p*-value 0.0000) was found for Fe (Table 4).

The correlations between trace-element levels in the coats of Hucul horses are summarised in Table 4. A strong positive correlation (R_s_ > 0.7) was found for the following pairs of trace elements in the coats of horses kept at stable A: Mn–Fe and Al–Fe, and those kept at stable B: Fe–Mn, Fe–Al, and Mn–Al (Table 5).

The value of Spearman’s R correlation coefficient and the level of significance for trace elements in each of the seasons studied are summarised in Tables S1–S3 (Supplementary materials).

**Figure 2:**
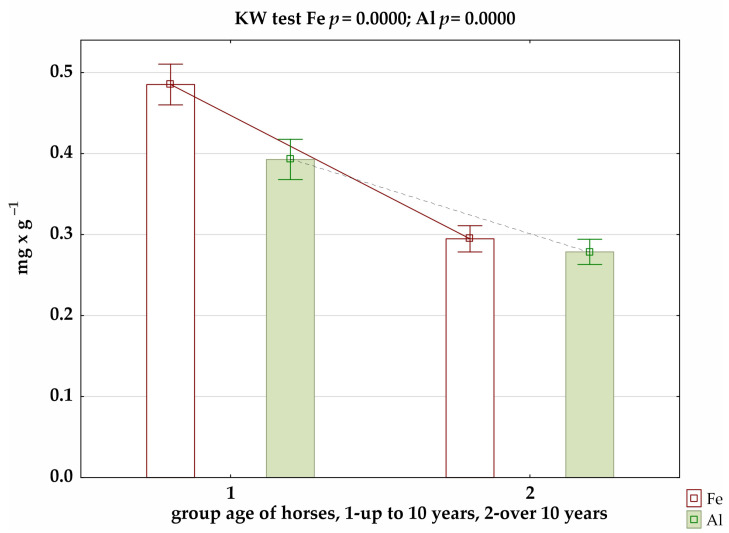
Level of selected micronutrients in relation to age of horses, where significance of differences was confirmed by Kruskal–Wallis test.

The results of the analysis of trace-element levels in the coats of Hucul horses in relation to age showed, for younger mares (up to 10 years old), significantly higher concentrations of Fe and Al (Figure 2). These differences were confirmed in the summer and winter seasons (Supplementary materials: Figures S1 and S2).

## 4. Discussion

Fresh and preserved grassland forage plays an important role in the nutrition of herbivores [22]. The quality of the forage depends, among other things, on the botanical composition of the area, weather conditions, maturity of the plants at the time the animal takes or harvests them, and drying or preservation method [23,24,25]. Undoubtedly, for the proper functioning of animal organisms, in addition to the proportion of basic components, the content of macro- and micronutrients and vitamins in the feed is important [4,26]. According to Meyer and Coenen [27], the iron content of the traditional feeds used in horse nutrition meets the needs of adult animals. Less absorption may occur if there is an excess of manganese in the feed. Iron, on the other hand, may be antagonistic to copper. In our study, the iron content was too low compared with the recommendations [27] in stable A hay and stable B haylage, whereas copper deficiencies were found in all feeds (Table 1). At the same time, high concentrations of manganese were found in the hay and haylage used in horse feed in both stables. Hoskin and Gee [22], when analysing the mineral composition of the pasture sward in one of the studs in New Zealand, determined Fe concentrations of 132–225 mg/kg DM. The micronutrient content of forage samples used in horse feed in Sweden and Norway [28] was higher for the minerals analysed, except for Cu (4.9 ± 1.61 mg.kg^−1^ dry mass), for which a higher value was recorded in our own study in green fodder at stable B (Table 1). The results of the analysis of the micronutrient content in forage samples in studies conducted in northern Europe showed similar levels [29,30,31]. In the studies conducted in New Zealand, slightly higher Cu and Mn contents were determined in pasture sward and almost twice as low Zn [22].

Numerous studies confirmed that the coat can be useful in assessing the mineral supply of the horse body [2,6,13,15,32]. Brummer-Holder et al. [33] found that trace-element concentrations in horse hair were higher than in blood. Moreover, animal hair is one of the most important matrices that can be used as a bioindicator of elements in the environment [11], similar to human hair [12]. However, herbivore coat is considered a better indicator of actual environmental pollution than human hair, as its diet consists almost exclusively of plants [34]. In a study by Fazio et al. [15], significantly lower concentrations of copper and zinc were found in the mane hair of horses kept in industrial areas than in our own study (Table 2). This may be due to the fact that mane and tail hairs have a constant growth rate that is close to linear, whereas the cyclic nature of coat growth is the mechanism by which animals change their coats to adapt to the seasons, allowing them to effectively thermo-regulate [2]. Therefore, it seems that the coat more realistically reflects the seasonal availability of elements to the body. In addition, in a study by Souza et al. [35], plasma and hair zinc concentrations were higher in non-industrial areas compared with samples taken in industrial areas. Stanek et al. [8] studied the effect of the addition of copper, zinc, and manganese chelates to the feed ration on the content of these elements in the hair coat of Polish pony foals raised in a stable or living in a reserve. They found that after 108 days of Cu supplementation in horses kept in stables, its concentration in the coat only slightly increased (from 7.26 to 8.03 mg kg^−1^ DM). In Polish Konik horses kept in a reserve, supplementation also contributed to an increase in Cu in the coat. According to Truchliński et al. [36], the normal level of copper in a horse’s coat is 13.6 mg kg^−1^. In our study, the level found was significantly lower (Table 2). In the study by Stanek et al. [8], zinc supplementation for 38 days in the Polish horse breed caused a significant increase in its level in the coat, whereas after 108 days, its concentration was similar to the control result, which may indicate adequate absorption and utilisation of this element by the body.

Our study showed differences in the levels of trace elements analysed in the coat of horses, which were statistically significant in both stables (Table 3). Topczewska obtained similar results in the analysis of the coats of Hucul horses [37], in whose study the concentrations of B, Ni, Si, Mo, I, and Cr in the coat also significantly differed depending on the season. Similarly, Souza et al. [35] showed differences between the summer and winter seasons, and significant differences were found for zinc concentrations in both hair and plasma. In a study by Biricik et al. [38], both seasonal changes and changes in diet had significant effects on mineral levels, but only in blood serum, not in horse hair. Given that the content of some elements in the coats of herbivores depends on their diets, seasonal differences seem logical. Madejón et al. [39] showed that, in autumn, there is a higher concentration of minerals in the green biomass due to the slower rate of plant growth than in spring. Primitive breeds are distinguished by very good forage utilisation, so periodic mineral deficiencies in forage and consequently in the animal’s body do not cause health disorders. Studies by Siwińska et al. [7] on the morphological structure of hair and mineral concentrations in Polish ponies (primitive breeds) showed significantly lower levels of minerals than in horses of other breeds. At the same time, the cited authors stated that the horses participating in the study were clinically healthy. Our own study used Hucul horses, such as primitive Polish Konik, for which the presence of slightly lower levels of trace elements does not cause health disorders.

Trace elements play a key role in normal growth, development, and numerous metabolic processes. Too low, but also too high, levels of them in the body can cause many dysfunctions and susceptibility to diseases without clinical signs [40]. Iron is extremely important not only for foals but also for horses in the early stages of training due to increased erythrocyte production. At the same time, an excess of this element can limit the absorption of phosphorus, copper, manganese, and zinc [27]. Copper is an essential element that acts as a cofactor for several enzymes involved in the antioxidant response, membrane and DNA integrity, and ATP production [40]. Copper deficiencies in foals can cause anaemia and pathological changes in the skeleton. According to Tirpák et al. [41], increased levels relative to normal physiological values of trace metals cause the production of reactive oxygen species in semen plasma. Deficiency of antioxidants that can inactivate free radicals results in the deterioration of sperm quality, and Cu deficiency, among others, results in impaired sperm motility parameters. In addition, deficiency can cause lameness with bone fractures, anaemia, and cardiovascular disorders. Abnormal zinc metabolism has resulted in osteochondrosis in horses [42]. In horses with allergic dermatitis, zinc levels were significantly lower than in healthy horses [43]. According to Paßlack et al. [44], zinc supplements are often used in equine diets to not only improve the quality of the skin and hoof but also increase immunity. However, high levels of zinc in the diet reduce the richness and fermentative activity of the equine gut microbiota. Oxidative stress, with changes in antioxidant trace-element levels, is a characteristic of respiratory diseases in draft horses [45]. The lower levels of trace-element concentrations found in the study horses were not associated with negative effects. The condition of the tested individuals was optimal, and the condition of the coat, mane, and tail hair was also normal. The mares regularly gave birth to offspring. Perhaps, as in the case of the Polish Konik breed [7], Hucul horses tolerate significantly lower concentrations of trace elements in the body, compared with other breeds, without health consequences.

Cygan-Szczegielniak [14] analysed the appearance of correlations between macro- and micronutrients in the coat, testes, liver, kidneys, and muscles of red deer but found statistically significant correlations only for the coat, which may confirm the fact that the coat is a good matrix for assessing the concentration of elements in the body. Sgorlon et al. [46], when analysing the concentration in the coat of dogs of many elements, found the occurrence of significant correlations between the content of Al and Fe, and in our study, an analogous relationship was established. Regardless of the season, a strong correlation was shown in stable A for the elements Fe–Mn and Al–Fe, and in stable B for Fe–Mn, Fe–Al, and Mn–Al. A strong positive correlation (R_s_ > 0.7) was shown for the following pairs of minerals during the summer season in the coat of horses in stable A: Fe–Mn, Fe–Al, C–-Zn, and Mn–Al, and in stable B: Fe–Al (Table S1). Similarly, a positive correlation (R_s_ > 0.7) was shown for Fe and Al during the winter season in the coat of the horses in stables A and B (Table S3). A Cygan-Szczegielniak study [14] found a strong positive correlation between K–Na (0.760; *p* ≤ 0.01) in the coat of red deer (*Cervus elaphus* L.). Kalashnikov et al. [47] analysed the effect of the mineral supply of trotter horses on their athletic performance and found that there was a negative correlation between the accumulation of elements, including Fe, Cu, Mn, and Al, and the speed of horses (r = −0.59). According to Długaszek [48], the key role in the correlations between elements is played by similarities in their chemical and physical properties, the life processes in which the elements participate, and the common environmental origins. At the same time, Cu–Zn and Fe–Cd are regarded the most frequently correlated elements. In our study, the correlation between Cu and Zn was confirmed. A very strong positive correlation was shown for Fe with Al in both stables: it was R_s_ = 0.9278 for stable A and R_s_ = 0.9472 for stable B, in both cases at a significance level of 0.0000 (Table 5).

Copper is an important component of many enzymes relevant for proper functioning of the body [49]. Effective absorption of Cu from feed may be adversely affected by Zn, as absorption of both elements follows a similar mechanism [50]. Zinc is important for normal reproduction. Lower concentrations of it have been found in cows that result in an abnormal luteal phase, delayed ovulation, and barrenness. Excess zinc in feed affects the metabolism of copper. In pigs, high zinc concentrations can cause bone fragility. An excessive supply of Zn was also found to alter the metabolism of Cu and Fe in chickens. Furthermore, the tibia bones of chickens retained less Fe and Cu in the liver and pancreas [42]. This is because Zn and Cu are regulated by the same metalloprotein, so they can mutually reduce the absorption and/or transfer of the other mineral [51]. Copper deficiency in herbivores may also be due to higher concentrations of sulphur and molybdenum in the feed. Pathologies associated with Fe, Cu, and Zn toxicity result in lipid damage to cell membranes [49]. The deficiency of trace elements involved in neutralizing free radicals can lead to oxidative stress. Although adequate Cu stores in the liver can induce metallothionein synthesis in enterocytes, Zn is the primary regulator of metallothionein synthesis, whereas Cu is a secondary stimulus. Metallothionein binds Zn, so it is not in a state of free ionization in the cell. At the same time, excess Zn-induced metallothionein in the diet also causes Cu sequestration within enterocytes. Cu deficiencies can also result in lower quality of colostrum. As Ha et al. [52] pointed out, Zn is not a major factor affecting Cu absorption in cows unless the diet contains at least two times the recommended amount of Zn. Although Fe-rich diets did not significantly alter Cu absorption in the gut, a high Fe content interferes with Cu availability during uptake by tissues such as the liver, resulting in clinical signs of Cu deficiency [52].

Our study also showed the effect of age on Fe and Al concentrations in the coats of horses (Figure 2). The results of the correlation analysis showed small and, at the same time, negative correlations for Fe, Cu, and Al. Furthermore, the study by Asano et al. [53], when analysing mineral concentrations in the mane hair of thoroughbred English racehorses aged 2 to 5 years, showed correlations between age and iron and manganese levels, but they were negative. This is confirmed by the results of our own study, as both Fe and Al were significantly lower in older individuals. In contrast, Brummer-Holder et al. [34] indicated a slight negative correlation (−0.39) between age and copper concentrations.

## 5. Conclusions

The study showed that the date of intake had a significant effect on the concentration of micronutrients analysed in the coat of Hucul horses. The highest concentration of Zn was found in the coat taken in summer. The coat taken in autumn was characterised by higher concentrations of Fe, Cu, Mn, and Al compared with the other seasons. The highest concentrations of Fe, Mn, and Al were found in the coat collected in winter, with the lowest levels of Zn. Positive correlations were found between the content of Fe and Mn, Fe and Al, and Mn and Al in the coat of Hucul horses. A clear inter-individual and inter-stable variability was found, which may indicate the need for further research that also takes into account other factors related to the mineral composition of the coat.

## Figures and Tables

**Table 1:** Content of selected trace elements in feeds used in horse nutrition, mean ± SD (mg·kg^−1^ DM).

	Green Fodder		*p*-Value	Hay		*p*-Value	Haylage		*p*-Value
	A	B		A	B		A	B	
Fe	47.3 ± 6.4	48.0 ± 2.3	ns	93.6 ± 12.1	73.4 ± 2.4	≤0.05	117.2 ± 41.4	137.7 ± 1.8	ns
Cu	4.0 ± 1.1	7.1 ± 0.8	≤0.05	3.2 ± 0.2	3.1 ± 0.1	ns	2.8 ± 1.1	2.6 ± 0.5	ns
Mn	98.5 ± 14.1	205.1 ± 41.0	≤0.05	200.5 ± 48.8	225.3 ± 31.8	≤0.05	171.8 ± 25.8	171.2 ± 7.9	ns
Zn	11.3 ± 2.6	20.3 ± 1.7	≤0.05	21.5 ± 3.6	14.3 ± 4.3	≤0.05	12.9 ± 2.9	11.6 ± 0.9	ns
Al	46.2 ± 4.5	39.9 ± 2.5	ns	36.4 ± 2.6	34.4 ± 1.8	ns	78.9 ± 28.2	141.6 ± 20.1	≤0.05

ns—Spearman R correlation coefficient value not statistically significant.

**Table 2:** Levels of selected micronutrients in coats of Hucul horses (mg·g^−1^).

Stud		Fe	Cu	Mn	Zn	Al
A	Mean ± SD	0.41 ± 0.21	0.007 ± 0.003	0.025 ± 0.02	0.11 ± 0.03	0.33 ± 0.18
	Range	0.06–0.80	0.00–0.02	0.00–0.083	0.07–0.22	0.06–0.75
B	Mean ± SD	0.31 ± 0.23	0.008 ± 0.004	0.022 ± 0.02	0.11 ± 0.03	0.30 ± 0.23
	Range	0.05–1.06	0.00–0.03	0.00–0.073	0.07–0.18	0.06 ± 1.17
	*p*-Value	0.0000	0.9823	0.0164	0.0639	0.0629
Total	Mean ± SD	0.35 ± 0.23	0.01 ± 0.003	0.02 ± 0.02	0.11 ± 0.03	0.31 ± 0.21

**Table 3:** Selected micronutrients in the coat of Hucul horses depending on the season (mean ± SD) (mg·g^−1^).

Stud	Season	Fe	Cu	Mn	Zn	Al
A	s	0.29 A ± 0.16	0.007 A ± 0.002	0.017 A ± 0.01	0.14 A ± 0.03	0.25 A ± 0.16
	a	0.44 B ± 0.24	0.006 B ± 0.001	0.019 AB ± 0.01	0.11 B ± 0.04	0.36 B ± 0.21
	w	0.49 B ± 0.16	0.008 C ± 0.004	0.029 B ± 0.02	0.09 B ± 0.01	0.38 B ± 0.16
*p*-Value		0.0000	0.0016	0.0014	0.0000	0.0002
B	s	0.18 A ± 0.10	0.008 A ± 0.002	0.014 A ± 0.01	0.15 B ± 0.01	0.18 A ± 0.12
	a	0.48 B ± 0.27	0.009 A ± 0.01	0.037 B ± 0.02	0.09 A ± 0.01	0.45 B ± 0.27
	w	0.25 A ± 0.18	0.005 B ± 0.002	0.022 C ± 0.01	0.09 A ± 0.02	0.25 A ± 0.17
*p*-Value		0.0000	0.0000	0.0000	0.0000	0.0000

s—Summer. a—Autumn. w—Winter; the means marked with different letters A, B, and C differ at *p* ≤ 0.001.

**Table 4:** Spearman rank correlation coefficient (R_s_) and *p*-value of trace elements in feed and coat.

Stable	Trace Element in		R_s_	*p*-Value
	Feed	Coat		
A	Fe	Fe	ns	0.519
	Cu	Cu	ns	0.873
	Mn	Mn	−0.2693	0.003
	Zn	Zn	0.2233	0.010
	Al	Al	ns	0.176
B	Fe	Fe	0.4818	0.0000
	Cu	Cu	−0.3983	0.0000
	Mn	Mn	−0.5655	0.0000
	Zn	Zn	−0.2079	0.011
	Al	Al	0.4784	0.0000

ns—Spearman R correlation coefficient value not statistically significant.

**Table 5:** R-Spearman correlation coefficients and *p*-value between micronutrients in the coat of Hucul horses overall, above diagonal for stable A, below for stable B.

	Fe	Cu	Mn	Zn	Al
Fe		0.3578 *p* = 0.0001	0.7037 *p* = 0.0000	−0.2351 *p* = 0.0118	0.9278 *p* = 0.0000
Cu	0.1667 *p* = 0.0481		0.3986 *p* = 0.0000	0.3382 *p* = 0.0002	0.2769 *p* = 0.0029
Mn	0.8062 *p* = 0.0000	ns		ns	ns
Zn	−0.3054 *p* = 0.0002	0.5711 *p* = 0.0000	−0.4686 *p* = 0.0000		−0.2538 *p* = 0.0064
Al	0.9472 *p* = 0.0000	ns	0.7692 *p* = 0.0000	−0.3564 *p* = 0.0000	

ns—Spearman R correlation coefficient value not statistically significant.

## Data Availability

The data that support the findings of this study are available from the corresponding author (J.T.) upon reasonable request.

## Supplementary Materials

The following supporting information can be downloaded at: https://www.mdpi.com/article/10.3390/ani12202770/s1, Figure S1: Level of selected micronutrients according to age of horses in summer season, for which the significance of differences was confirmed by Kruskal–Wallis test and Figure S2: Level of selected micronutrients according to age of horses in winter season, for which the significance of differences was confirmed by Kruskal–Wallis test; Table S1: R-Spearman correlation coefficients and *p*-value between micronutrients in the coat of Hucul horses in summer season (s), above the diagonal for stable A and below for stable B; Table S2: R-Spearman correlation coefficients and *p*-value between micronutrients in the coat of Hucul horses in autumn season (a), above the diagonal for stable A and below for stable B and Table S3: R-Spearman correlation coefficients and *p*-value between micronutrients in the coat of Hucul horses in autumn season (a), above the diagonal for stable A and below for stable B.
