# Supplementary material for: Seasonal Changes in Trace-Element Content in the Coat of Hucul Horses
Source: Animals (Basel). 2022 Oct 14;12(20):2770. doi: 10.3390/ani12202770 (PMC9597826; doi:10.3390/ani12202770)
Supplement: Supplementary file 1 [file animals-12-02770-s001.zip › Supplementary material Tables S1-S3.pdf]

Table S1. R-Spearman correlation coefficients and p-value between micronutrients in the coat of Hucul horses in the summer season (s), above the diagonal for stable A and below for stable B.

	Fe	Cu	Mn	Zn	Al
Fe		0.4550<br>p=0.0036	0.8060<br>p=0.0000	ns	0.9699<br>p=0.0000
Cu	0.4550<br>p=0.0036		0.5194<br>p=0.0007	0.7066<br>p=0.0000	0.4377<br>p=0.0053
Mn	0.5874<br>p=0.0000	ns		0.6212<br>p=0.0000	0.8243<br>p=0.0000
Zn	ns	0.4653<br>p=0.0013	ns		0.3195<br>p=0.0474
Al	0.9419<br>p=0.0000	ns	0.6467<br>p=0.0000	0.5666<br>p=0.0000	

ns – Spearman correlation coefficient value not statistically significant.

Table S2. R-Spearman correlation coefficients and p value between micronutrients in the coat of Hucul horses in the autumn season (a), above the diagonal for stable A and below for stable B.

	Fe	Cu	Mn	Zn	Al
Fe		0.4690<br>p=0.0039	0.8118<br>p=0.000	ns	0.8792<br>p=0.0000
Cu	0.5221<br>p=0.0001		0.4219<br>p=0.0104	0.4009<br>p=0.0154	0.4294<br>p=0.0090
Mn	0.8493<br>p=0.0000	ns		ns	ns
Zn	ns	0.5132<br>p=0.0002	ns		0.6789<br>p=0.0000
Al	0.8513<br>p=0.0000	0.3645<br>p=0.0109	0.6356<br>p=0.0000	ns	

ns – Spearman correlation coefficient value not statistically significant.

Table S3. R-Spearman correlation coefficients and p value between micronutrients in the coat of Hucul horses in the autumn season (a), above the diagonal for stable A and below for stable B.

	Fe	Cu	Mn	Zn	Al
Fe		0.4284<br>p=0.0065	ns	ns	0.8318<br>p=0.0000
Cu	ns		0.3927<br>p=0.0134	ns	ns
Mn	0.7482<br>p=0.0000	ns		-0.3557<br>p=0.0263	ns
Zn	ns	0.6732<br>p=0.0000	ns		ns
Al	0.9015<br>p=0.0000	ns	0.6720<br>p=0.0000	ns	

ns – Spearman correlation coefficient value not statistically significant.
