# Supplementary material for: Seasonal Changes in Trace-Element Content in the Coat of Hucul Horses
Source: Animals (Basel). 2022 Oct 14;12(20):2770. doi: 10.3390/ani12202770 (PMC9597826; doi:10.3390/ani12202770)
Supplement: Supplementary file 1 [file animals-12-02770-s001.zip › Supplementary materials Figures S1-S2.pdf]

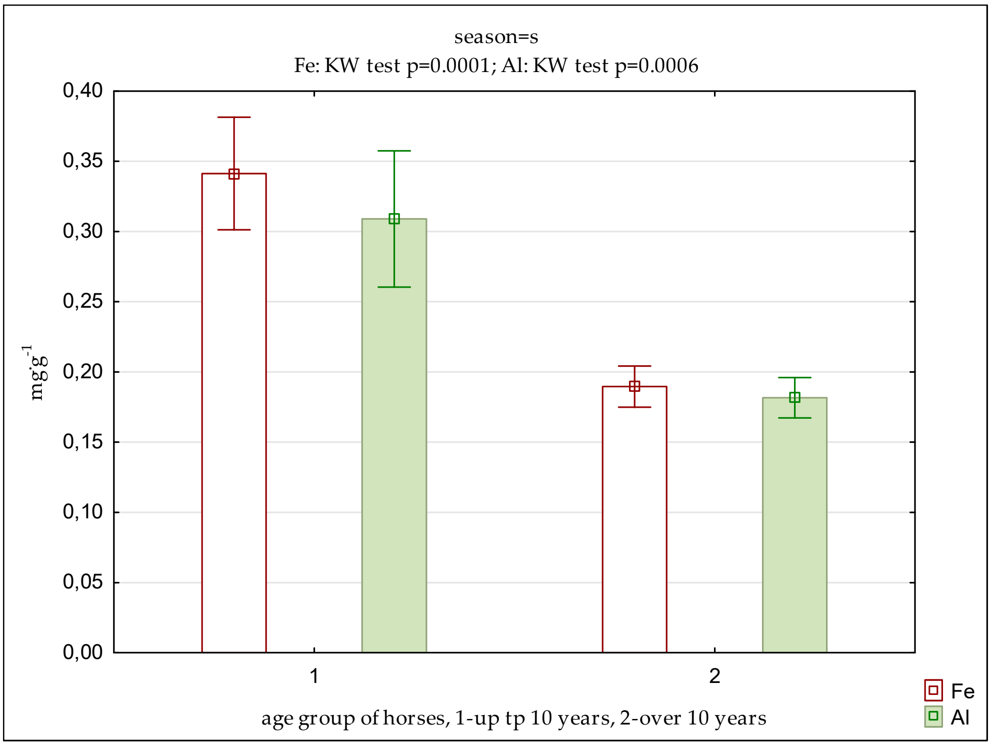

Figure S1. The level of selected micronutrients according to the age of the horses in the summer season, for which the significance of differences was confirmed by the Kruskal-Wallis test.

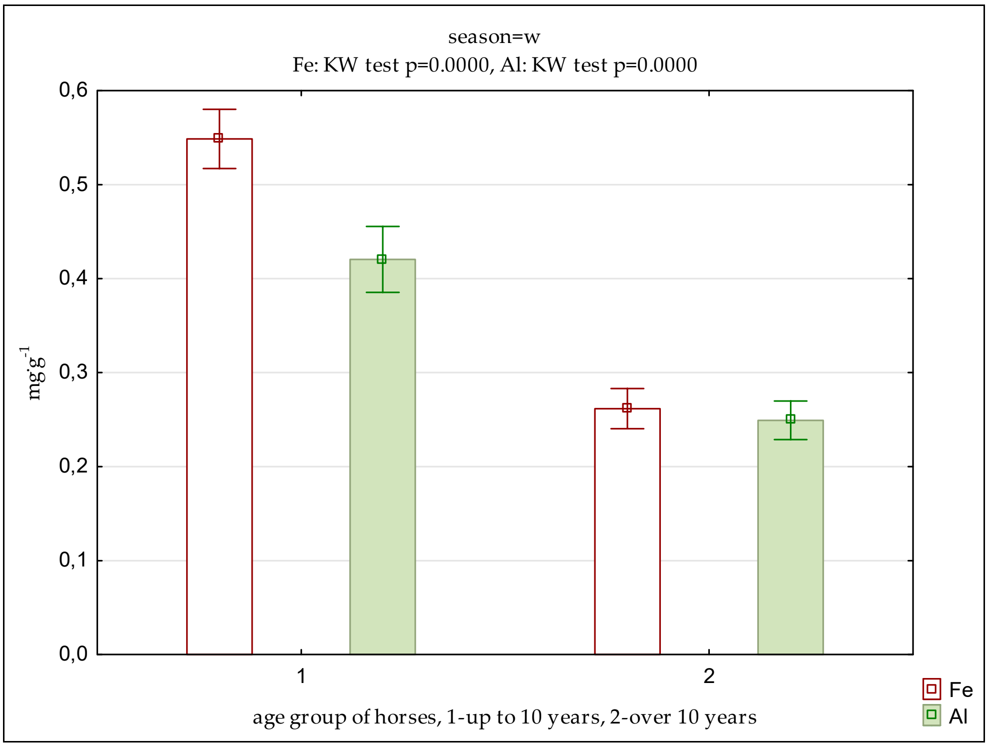

Figure S2. The level of selected micronutrients according to the age of the horses in the winter season, for which the significance of differences was confirmed by the Kruskal-Wallis test.
